# Dengue Dynamics in Binh Thuan Province, Southern Vietnam: Periodicity, Synchronicity and Climate Variability

**DOI:** 10.1371/journal.pntd.0000747

**Published:** 2010-07-13

**Authors:** Khoa T. D. Thai, Bernard Cazelles, Nam Van Nguyen, Long Thi Vo, Maciej F. Boni, Jeremy Farrar, Cameron P. Simmons, H. Rogier van Doorn, Peter J. de Vries

**Affiliations:** 1 Division of Infectious Diseases, Tropical Medicine and AIDS, Academic Medical Center, Amsterdam, the Netherlands; 2 Center for Infection and Immunity (CINIMA), Academic Medical Center, University of Amsterdam, Amsterdam, the Netherlands; 3 Oxford University Clinical Research Unit, Hospital for Tropical Diseases, Ho Chi Minh City, Vietnam; 4 UMR 7625, UPMC-CNRS-ENS, Ecole Normale Supérieure, Paris, France; 5 UMI 209, IRD-UPMC, Bondy, France; 6 Binh Thuan Medical College, Phan Thiet City, Vietnam; 7 Center for Tropical Medicine, Nuffield Department of Clinical Medicine, University of Oxford, Center for Clinical Vaccinology and Tropical Medicine, Oxford, United Kingdom; 8 MRC Centre for Genomics and Global Health, University of Oxford, Oxford, United Kingdom; Duke University-National University of Singapore, Singapore

## Abstract

**Background:**

Dengue is a major global public health problem with increasing incidence and geographic spread. The epidemiology is complex with long inter-epidemic intervals and endemic with seasonal fluctuations. This study was initiated to investigate dengue transmission dynamics in Binh Thuan province, southern Vietnam.

**Methodology:**

Wavelet analyses were performed on time series of monthly notified dengue cases from January 1994 to June 2009 (i) to detect and quantify dengue periodicity, (ii) to describe synchrony patterns in both time and space, (iii) to investigate the spatio-temporal waves and (iv) to associate the relationship between dengue incidence and El Niño-Southern Oscillation (ENSO) indices in Binh Thuan province, southern Vietnam.

**Principal Findings:**

We demonstrate a continuous annual mode of oscillation and a multi-annual cycle of around 2–3-years was solely observed from 1996–2001. Synchrony in time and between districts was detected for both the annual and 2–3-year cycle. Phase differences used to describe the spatio-temporal patterns suggested that the seasonal wave of infection was either synchronous among all districts or moving away from Phan Thiet district. The 2–3-year periodic wave was moving towards, rather than away from Phan Thiet district. A strong non-stationary association between ENSO indices and climate variables with dengue incidence in the 2–3-year periodic band was found.

**Conclusions:**

A multi-annual mode of oscillation was observed and these 2–3-year waves of infection probably started outside Binh Thuan province. Associations with climatic variables were observed with dengue incidence. Here, we have provided insight in dengue population transmission dynamics over the past 14.5 years. Further studies on an extensive time series dataset are needed to test the hypothesis that epidemics emanate from larger cities in southern Vietnam.

## Author Summary

Dengue has become a major international public health problem due to increasing geographic distribution and a transition from epidemic transmission with long inter-epidemic intervals to endemic transmission with seasonal fluctuation. Seasonal and multi-annual cycles in dengue incidence vary over time and space. We performed wavelet analyses on time series of monthly notified dengue cases in Binh Thuan province, southern Vietnam, from January 1994 to June 2009. We observed a continuous annual mode of oscillation with a non-stationary 2–3-year multi-annual cycle. We used phase differences to describe the spatio-temporal patterns which suggest that the seasonal wave of infection was either synchronous with all districts or moving away from Phan Thiet district, while the multi-annual wave of infection was moving towards Phan Thiet district. We also found a strong non-stationary association between ENSO indices and climate variables with dengue incidence. We provided insight in dengue population transmission dynamics over the past 14.5 years. Further studies on an extensive time series dataset are needed to test the hypothesis that epidemics emanate from larger cities in southern Vietnam.

## Introduction

Recent estimates indicate that approximately 3.5 billion people, about 55% of the world's population, live in countries at risk for dengue infection [1]. Dengue ranks among the most important infectious diseases in many countries in the tropics and subtropics. Dengue virus (DENV) transmission occurs primarily through bites by the mosquito vectors, *Aedes aegypti*, which feed preferentially on human blood, and are often found in and around human dwellings [2], [3]. Infection with any of the four dengue serotypes results in either asymptomatic infection, or a spectrum of clinically apparent disease ranging from mild undifferentiated febrile illness to severe dengue of which dengue shock syndrome (DSS) is the most common life threatening syndrome [4]. Dengue has become a major international public health problem due to increasing geographic distribution and a transition from epidemic transmission with long inter-epidemic intervals to endemic with seasonal fluctuation [5], [6]. Seasonal and multi-annual cycles in dengue incidences vary over time and space [7].

Multiple factors may influence the dynamics of dengue including environmental and climate factors, host-vector interactions and the population-wide immune landscape [8]. Climate variability is postulated to be an important determinant of dengue epidemics [9], [10]. Meteorological conditions (temperature, humidity, wind and precipitation) may directly or indirectly affect vector survival, life-span, development and reproductive rates which could influence dengue spatio-temporal oscillations [11]. Dengue incidence time series are characterized by nonlinear dynamics, with strong seasonality, multi-annual oscillations and non-stationary temporal variations (i.e. irregular temporal fluctuations in incidence) [12]. These features complicate the detection of both temporal and spatial patterns. Therefore, conventional methods such as Fourier analysis and generalized linear models (GLM) may be inadequate.

Wavelet analysis is suitable for investigating time series data from non-stationary systems and for inferring associations within such systems. Wavelet analysis is able to measure associations (coherency) between two time-series at any frequency (period) band and at every time-window period. Wavelet coherency analyses have been used to compare time series of disease incidence across localities and countries for the characterization of the evolution of epidemics periodicity and the identification of synchrony. Wavelet analyses have been used in analyzing various human infectious disease dynamics such as measles, influenza, leishmaniasis and dengue [7], [13]–[17].

Phase analysis completes wavelet analysis by allowing the investigation of phase shift between epidemics at different locations. For instance, phase differences at a given period and time window allowed the observations of spatio-temporal patterns of measles infections prior to measles vaccination [15]. Phase angles illustrated spatio-temporal waves of measles and dengue infection which showed that waves originated from regional population centers [15], [18].

In this study, we performed wavelet analysis on time series of notified dengue cases (i) to detect and quantify variability in dengue incidence and how it progresses through time, (ii) to describe synchrony patterns in both time and space, (iii) to investigate the spatio-temporal waves of infection and (iv) to associate the relationship between dengue incidence and El Niño-Southern Oscillation (ENSO) and local climate variables in Binh Thuan province, southern Vietnam.

## Methods

### Epidemiological and climatic data

Binh Thuan Province is located along the south-eastern coast of Vietnam, 150 km northeast of Ho Chi Minh City. It covers 7,828 km^2^ and the population from census data was 1,032,993 inhabitants in 1999 [19]. Binh Thuan Province is divided into 122 administrative units including 97 communities in semi-rural areas, 14 wards in Phan Thiet City (the capital), and 11 small towns and nine districts, including Phu Quy – an island off the coast.

Monthly notified dengue cases (i.e. clinically suspected cases of dengue without laboratory confirmation) in nine districts in Binh Thuan Province, southern Vietnam, were obtained from the provincial prevention department. Data include cases from January 1995 through June 2009. The provincial meteorological department provided historical monthly climatic data (i.e. mean temperature, humidity and rainfall). The ENSO indices (i.e. Multivariate El Niño-Southern Oscillation Index (MEI), NIÑO 1+2, NIÑO 3, NIÑO 4 and NIÑO 3.4) were extracted from http://www.cpc.ncep.noaa.gov/data/indices/.

### Wavelet analysis – periodicity of dengue

Wavelet analysis was performed to explore the periodicity in the dengue incidence time series [12], [13]. Wavelet analysis provides the possibility of investigating and quantifying the temporal evolution of time series with different rhythmic components. In addition, wavelet analysis allows detection of changes in periodicity in time. The Morlet wavelet was used and all analyses were performed with Matlab software [13]. All dengue incidence time series were square root transformed and all series were normalized and the trend was suppressed before analyses. The trend was suppressed before analysis by removing of the periodic components with period components greater than 8 years by using a classical low pass filter.

### Coherency and phase analyses – synchrony between districts and climate variability

To quantify the dependencies between two time series, wavelet coherence analyses in neighboring districts were performed. Wavelet coherence allows checking if different periodic modes of two time series tend to oscillate simultaneously, rising and falling together and quantifying the synchrony of these two time series. In addition, phase analysis was used to characterize the association between these two time series [13]. All significance levels were based on 1000 bootstrapped series.

### Spatio-temporal patterns

To investigate spatio-temporal patterns of dengue dynamics, phase difference was calculated between epidemics at different districts relative to Phan Thiet district, in which Phan Thiet City is the capital. Phase analyses generate a phase angle at each time step at a given periodic band. Further, we compute the phase difference to analyze the time delay between dengue incidences at different locations.

## Results

### Periodicity of dengue

**Figure 1:**
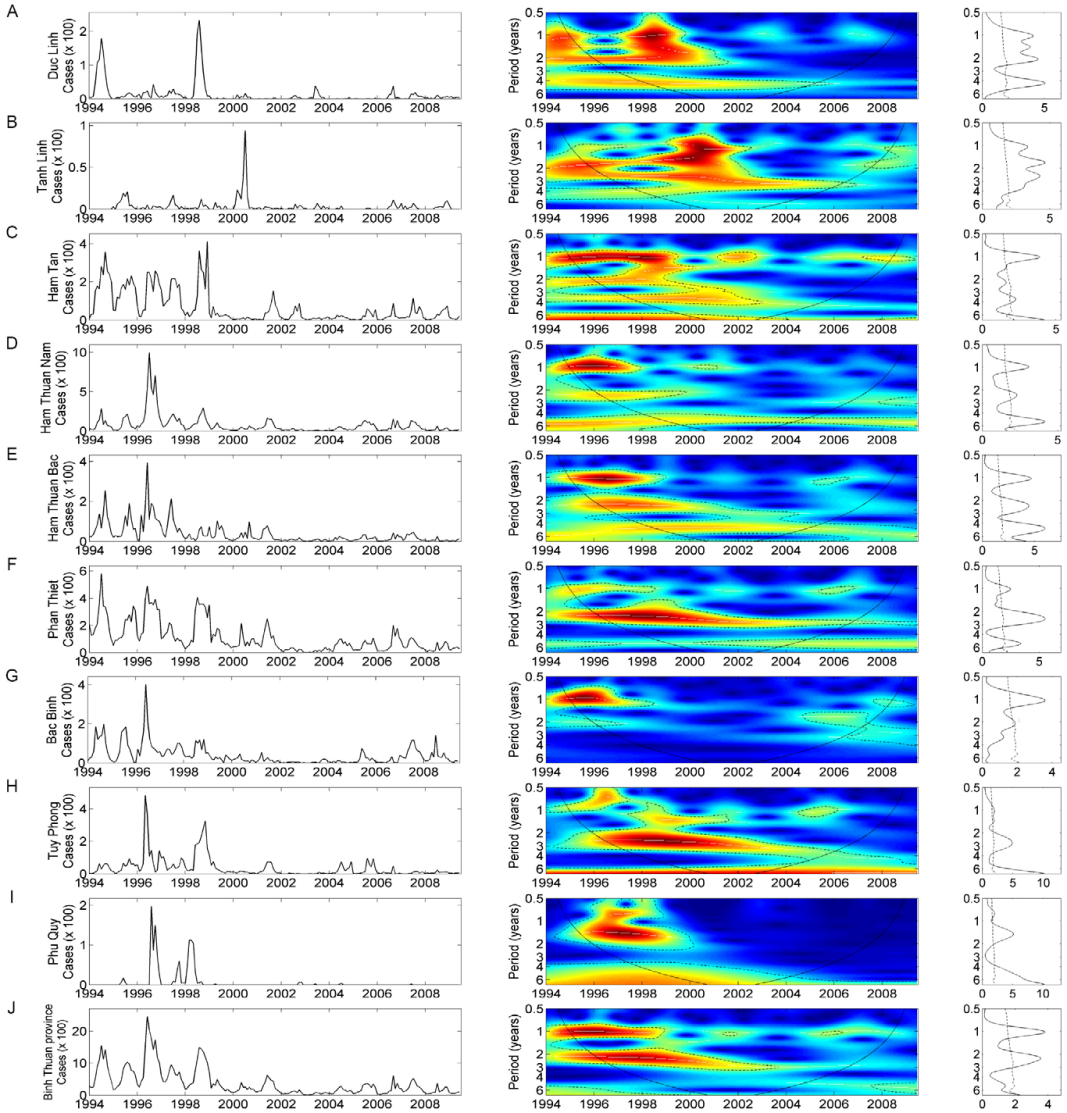
Wavelet analyses of dengue time series with monthly data from 1994 to 2009 in 9 districts of Binh Thuan province. (A) Duc Linh, (B) Tanh Linh, (C) Ham Tan, (D) Ham Thuan Nam, (E) Ham Thuan Bac, (F) Phan Thiet, (G) Bac Binh, (H) Tuy Phong, (I) Phu Quy, (J) Binh Thuan province. For each district (i) left panel: the time series of cases in the district (ii) middle panel: the wavelet power spectrum of dengue cases (square rooted and normalized and trend suppressed); colors code for increasing spectrum intensity, from blue to red; dotted lines show statistically significant area (threshold of 5% confidence interval); the black curve delimits the cone of influence (region not influenced by edge effects (iii) right panel: to the mean spectrum (solid line) with its threshold value of 5% (dotted line).

Wavelet analyses of time series data from nine districts of Binh Thuan province are displayed in Figure 1. In general, the mean spectra show periodicity for all districts. More specifically, periodicities were detected in the 1-year and the 2–3-year bands. However, dengue dynamics showed different evolutions across the nine time series which can be divided into three groups based on wavelet cluster analysis [20]: (i) the first group consists of three districts (Figure 1-F, H and I) in which a multi-annual cycle was predominant and the annual cycle was weak. Phan Thiet and Tuy Phong district showed a constant cycle of between 2–3-years from 1996–2001 (Figure 1-F and H). Tanh Linh (Figure 1-B) has similar dynamics to the districts of the first group, but with a less marked seasonality; (ii) in three districts, the annual cycle was predominant (Figure 1-D, E and G); (iii) a third group consists of districts in which both annual and multi-annual cycle were present (Figure 1-A and C). The districts in this last group were also dominated by large epidemics in 1994 and 1998. The wavelet cluster tree is shown in Figure S1. When all time series were aggregated, the oscillation of dengue incidence showed a strong annual mode of oscillation with a significant transient multi-annual mode of oscillation around a 2–3-year periodic band between 1996–2001 (Figure 1J).

### Synchrony between districts

**Figure 2:**
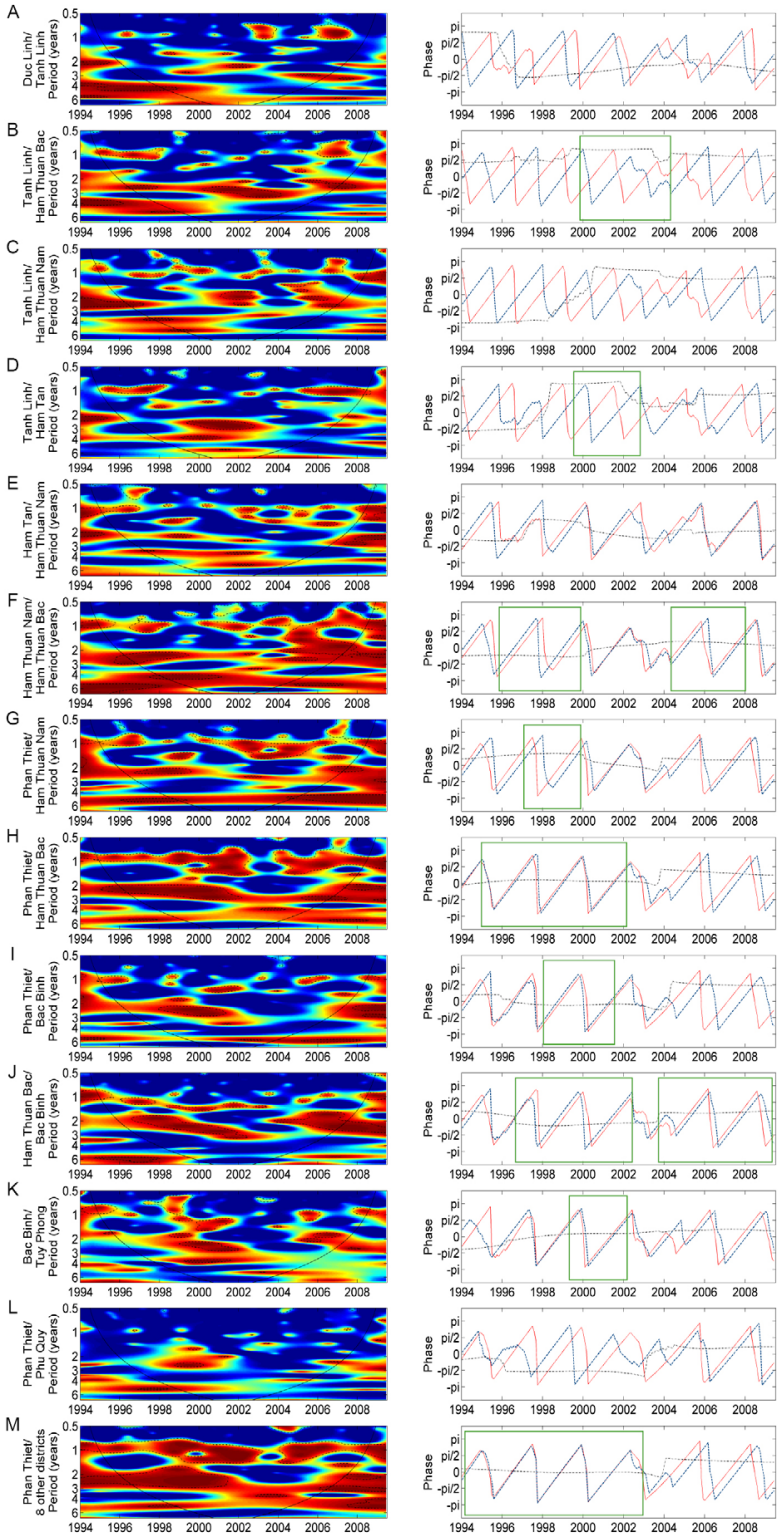
Wavelet coherence and phase analyses of dengue time series between neighboring districts in Binh Thuan province. The left panel represents the wavelet coherence. Blue, low coherence; red, high coherence. The dotted lines show α = 5% significance level. The cone of influence (black curve) indicates the region not influenced by edge effects. The right panels represent the phase analyses between two districts (in blue and red), based on wavelets for 2–3-year periodic band. Green boxes represents the period of time where coherency is significant, when interpretation of analysis was possible. Red lines: first district; blue lines: second district; dashed black lines: phase difference between the two oscillating components.

We compared dengue dynamics between neighboring districts when periodicities were detected in each district (Figure 2-A to L). High coherencies in all comparisons were detected for the 1-year periodicity (left). This annual mode of oscillations was also observed in the phase angle analyses (see Figure S2). Multi-annual oscillations at a 2–3-year period were identified, although these oscillations were transient and varied in time and space. There was a tendency that the multi-annual oscillation returned after 2006. Globally, coherency analysis between dengue incidence time series in Phan Thiet district and the eight other districts together shows two main regions of high significant coherence (Figure 2M). The first one is for the 1 year periodic band in 1995–2003 and in 2004–2008 and the second is for the 2–3-years bands in 1996–2001. Although there is a third significant coherence region (4 year periodic band, 2003–2004), it must be interpreted with caution due to the short length of the time series.

### Spatio-temporal patterns in dengue dynamics

**Figure 3:**
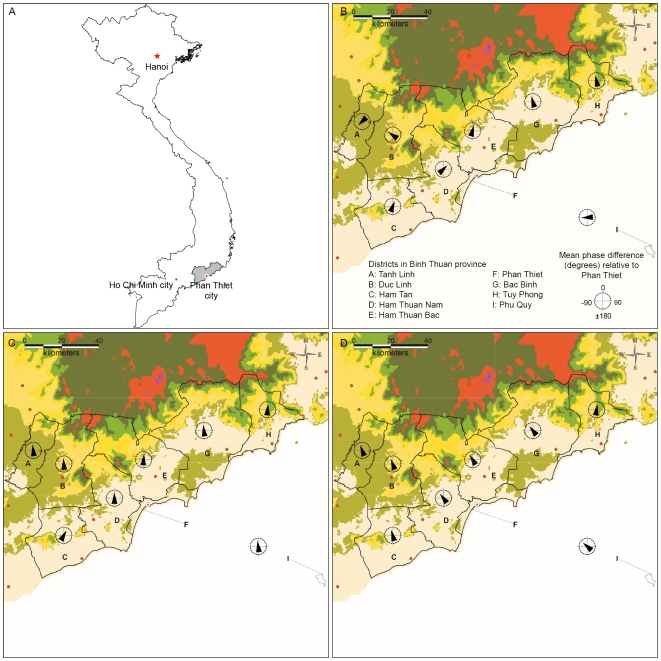
Phase differences from Phan Thiet district. (A) Map of Vietnam (B) Mean phase differences for 1996–2001 data at the 2–3-year period band. (C) Mean phase differences for 1995–2002 data at the 1-year period band. (D) as in C, but for 2004–2008 data.

Spatio-temporal patterns were investigated by calculating phase differences as published previously [15]. Based on the results in Figure 2M, phase differences were calculated for three periods: between 1996 and 2001, when the 2–3-year periodic band was pronounced; from 1995 to 2002 and from 2004 to 2008, when the annual periodic band was significant. The average phase difference relative to Phan Thiet is shown in Figure 3. Data indicate that the multi-annual (2–3-year period band) wave of dengue infection was moving away from another epicenter, rather than from Phan Thiet district as only three districts lagged behind (negative phase difference) or the time delay was greater than a half cycle. In contrast, the annual wave of dengue infection was either synchronous (1995–2002) with all districts or moving away (2004–2008) from Phan Thiet district.

### Association between dengue incidence and climate variability

**Figure 4:**
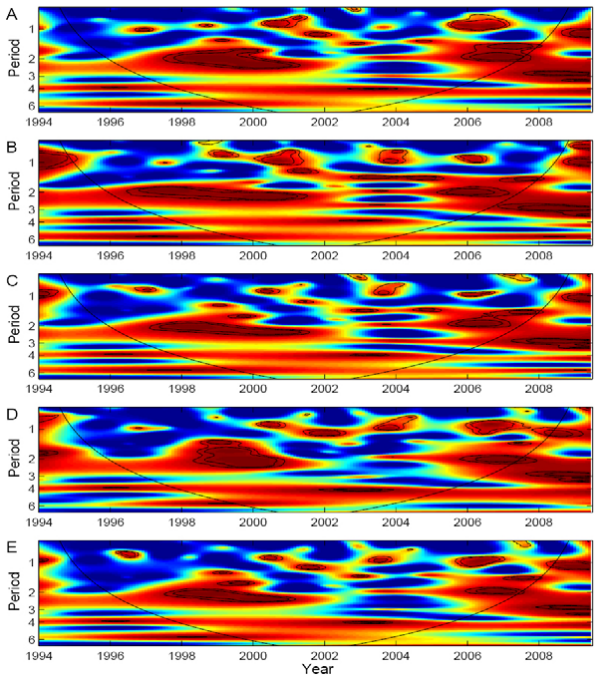
Wavelet coherence analyses of dengue incidence with ENSO indices. (A) MEI, (B) NIÑO 1+2, (C) NIÑO 3, (D) NIÑO 4 and (E) NIÑO 3.4. Blue, low coherence; red, high coherence. The dotted lines show α = 5% and 10% significance levels. The cone of influence (black curve) indicates the region not influenced by edge effects.

**Figure 5:**
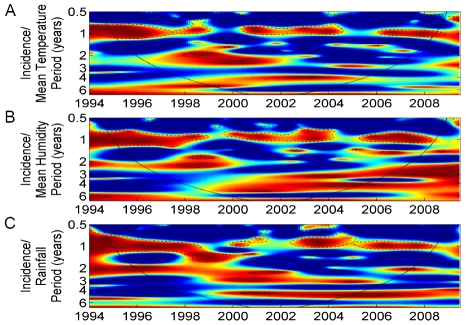
Wavelet coherence analyses of dengue incidence with local climate variables. (A) mean temperature, (B) humidity and (C) rainfall. Blue, low coherence; red, high coherence. The dotted lines show α = 5% significance levels. The cone of influence (black curve) indicates the region not influenced by edge effects.

For climate association analyses, the aggregated dengue incidence time series from all districts (Figure 1J, left) was used. Wavelet coherency between dengue incidence and ENSO indices and climate variables are shown in Figure 4 and 5, respectively. ENSO indices and climate variables were significantly associated with dengue incidence in the 2–3-year periodic band, although the associations were transient in time.

## Discussion

In this study, we have demonstrated annual modes of oscillation with a significant multi-annual cycle around a 2–3-year periodic band from 1996–2001. Synchrony in time and space for the annual cycle was detected and an overall synchronous 2–3-year cycle was quantified between Phan Thiet district and the other districts. We described the spatio-temporal patterns by using mean phase differences which suggest that the seasonal wave of infection was synchronous or moving away from Phan Thiet district. This contrasts the 2–3-year wave of infection, which was moving away from another epicenter towards Phan Thiet district. A significant non-stationary association between ENSO indices with dengue incidence in the multi-annual cycle was observed.

The ubiquitous periodicity of dengue incidence in Binh Thuan province is in accordance with dengue dynamics in Thailand [7], [18]. Annual periodic patterns are a common phenomenon in dengue transmission and have been reported in many tropical and subtropical countries, but the identification of a periodic multi-annual (e.g. 2–3-year) cycle differs between countries and in analyses used. A multi-annual periodicity of 3 years was detected in DHF data from Thailand using spectral density analysis [21], while recently, multi-annual dengue periodicity was not shown explicitly in reported dengue cases from Puerto Rico, Mexico and Thailand using wavelet analysis [16]. Compared to our analyses, Johansson *et al.* [16] have log-transformed their data and used a null hypothesis assuming that sequential observations are dependent and accounted for the potential influence of short-term autocorrelation on long-term time series. The use of log-transformation has homogenized the variance of the time series which provided more weight to the dominant annual component. Whether the multi-annual periodicity of dengue incidence exists or not, the detection of periodic cycles for dengue incidence itself provides no insights into the processes that cause dengue incidence oscillations (e.g. environmental and climate factors, host-vector interactions and population herd immunity). Interactions between intrinsic and extrinsic factors remain important to be elucidated and need further exploration [22].

Displacement of the predominant serotype has been associated with outbreaks and multi-annual fluctuations may be due to antibody-dependent enhancement (ADE) of infection with heterologous serotypes [23]–[25]. Other factors that may be important for the occurrence of multi-annual cycles may be the impact of population movement, demographic change and climatic variables [11]. Our findings provide evidence for a non-stationary relationship between ENSO indices and dengue incidence in the 2–3-year periodic cycle and associations with climate variables were significantly present. This suggests that El Niño affects local climatic variables which in turn affect dengue incidence in Binh Thuan province. However, it seems that large-scale indices seem to predict ecological processes better than local weather which has been addressed previously [26], [27].

Our results also show a large decrease in the variability of dengue incidence after 2000–2001 corroborating the Thailand dataset [28]. Possible explanations for the observed decrease could be a modification of the climatic conditions, a reduction in transmission due to declining mosquito populations, declining contact between human and mosquito populations, and/or modifications in diagnosis, classification and reporting dengue cases. A demographic shift to lower birth and death rates could induce modifications to dengue oscillations, a possible explanation which needs to be tested in Vietnam [28].

We have examined the spatio-temporal patterns of dengue infection, by computing the phase differences. Interestingly, mean phase differences suggested that the multi-annual wave of dengue infection was moving towards to Phan Thiet district and might originate from another, but nearby epicenter. In contrast, the annual wave of dengue infection was either synchronous with all districts or the wave of infection initiated in Phan Thiet district. Regional or local transmission in Phan Thiet district, probably influenced by climatic phenomena, modulated the annual cycles. Similar to the spatio-temporally travelling waves in incidence of DHF from Thailand, the multi-annual cycles in Vietnam may emanate from larger cities (e.g. Ho Chi Minh City which is located 200 km to the south west or Hanoi, the capital in the north of Vietnam) [18]. Further extensive analyses on surveillance data from other regions are needed to test this hypothesis.

Wavelet analyses have revealed interesting information on dengue transmission dynamics in Binh Thuan province. However, there was a limitation in the present study. Dengue data used in this study were based on notified clinically-suspected dengue cases from hospitals or clinics without laboratory confirmation. These numbers may be an underestimation of the true incidence [29], [30]. Notification behavior and clinical suspicion are both subjective parameters which may be influenced by communications with colleagues, media attention, and history of clinicians on reporting. To mitigate these reasonable limitations, Vietnam has consistent reporting of dengue cases and clinicians are well trained in recognizing dengue, both of which indicate that surveillance data would be a good representation of symptomatic dengue patients. Another limiting factor is that the circulating serotypes were not known during the whole time period. Although, the dengue multi-annual cycle oscillates around a 2–3-year periodic band, the cycle for all serotypes may differ from each other resulting in different dengue serotype multi-annual cycles.

In conclusion, we observed a strong annual mode of oscillation with a significant multi-annual cycle around a 2–3-year periodic band from 1996–2001. Multi-annual waves of infection probably started outside Binh Thuan province. We report a significant non-stationary association between ENSO indices with dengue incidence for the multi-annual cycle. The existence of these plausible climatic determinants may be particularly interesting in the context of the development of early warning systems in which reliable predictors of dengue epidemics may be usefully employed. Nevertheless this requires empirical proof and the understanding of the mechanisms linking climatic conditions and dengue propagation. Our findings provide insights into the long-term persistence and spatial spread of dengue throughout Binh Thuan province, southern Vietnam. Further studies on a more extensive time series dataset of a larger area could shed more light onto the spatio-temporal patterns in Vietnam.

## Supporting Information

Figure S1 Cluster tree of the dengue time series. The cluster tree was obtained by applying a classification method with a covariance threshold at C = 99% of the total covariance as described by Rouyer et al. (A) Duc Linh, (B) Tanh Linh, (C) Ham Tan, (D) Ham Thuan Nam, (E) Ham Thuan Bac, (F) Phan Thiet, (G) Bac Binh, (H) Tuy Phong, (I) Phu Quy.
(0.06 MB TIF)

Figure S2 Phase analyses of dengue time series between neighboring districts in Binh Thuan province. Phase analyses between two districts (in blue and red), based on wavelets for 1-y periodic band.
(1.21 MB TIF)
