# Supplementary figures and images for: Dengue Dynamics in Binh Thuan Province, Southern Vietnam: Periodicity, Synchronicity and Climate Variability
Source: PLoS Negl Trop Dis. 2010 Jul 13;4(7):e747. doi: 10.1371/journal.pntd.0000747 (PMC2903474; doi:10.1371/journal.pntd.0000747)

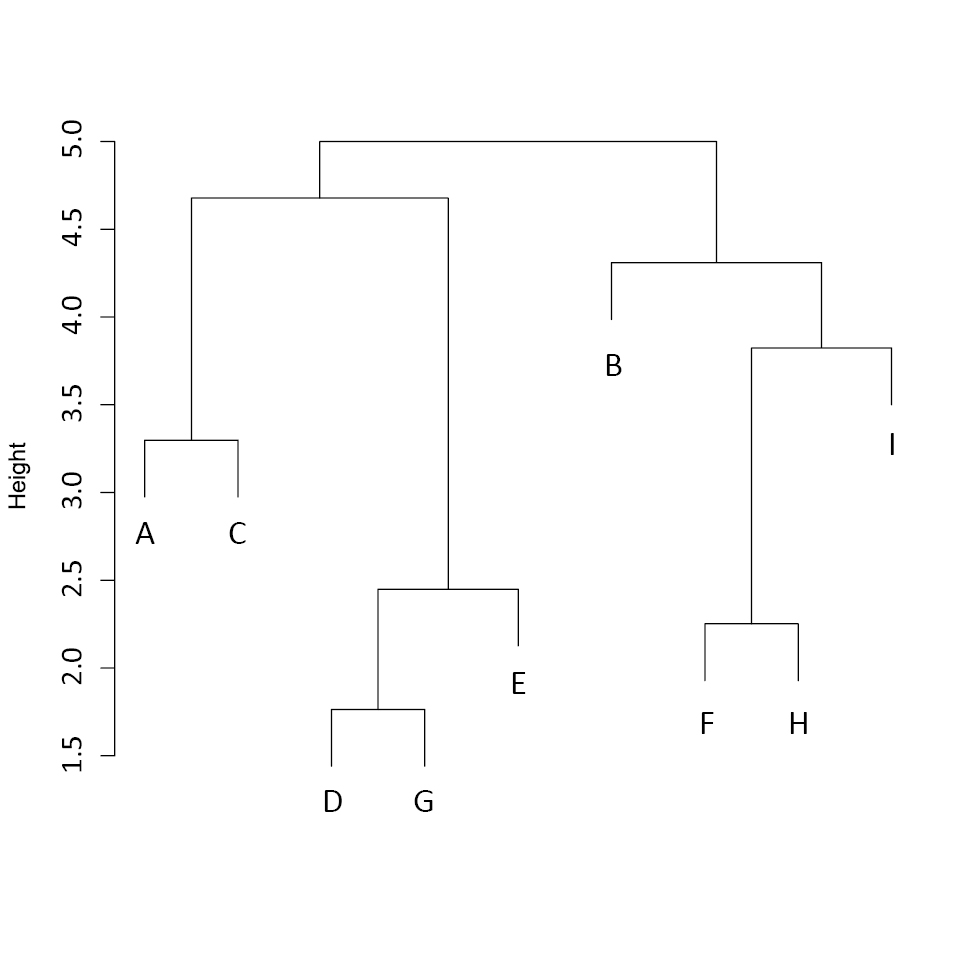

Supplement: Figure S1 — Cluster tree of the dengue time series. The cluster tree was obtained by applying a classification method with a covariance threshold at C = 99% of the total covariance as described by Rouyer et al. (A) Duc Linh, (B) Tanh Linh, (C) Ham Tan, (D) Ham Thuan Nam, (E) Ham Thuan Bac, (F) Phan Thiet, (G) Bac Binh, (H) Tuy Phong, (I) Phu Quy. (0.06 MB TIF) [file pntd.0000747.s001.tif]

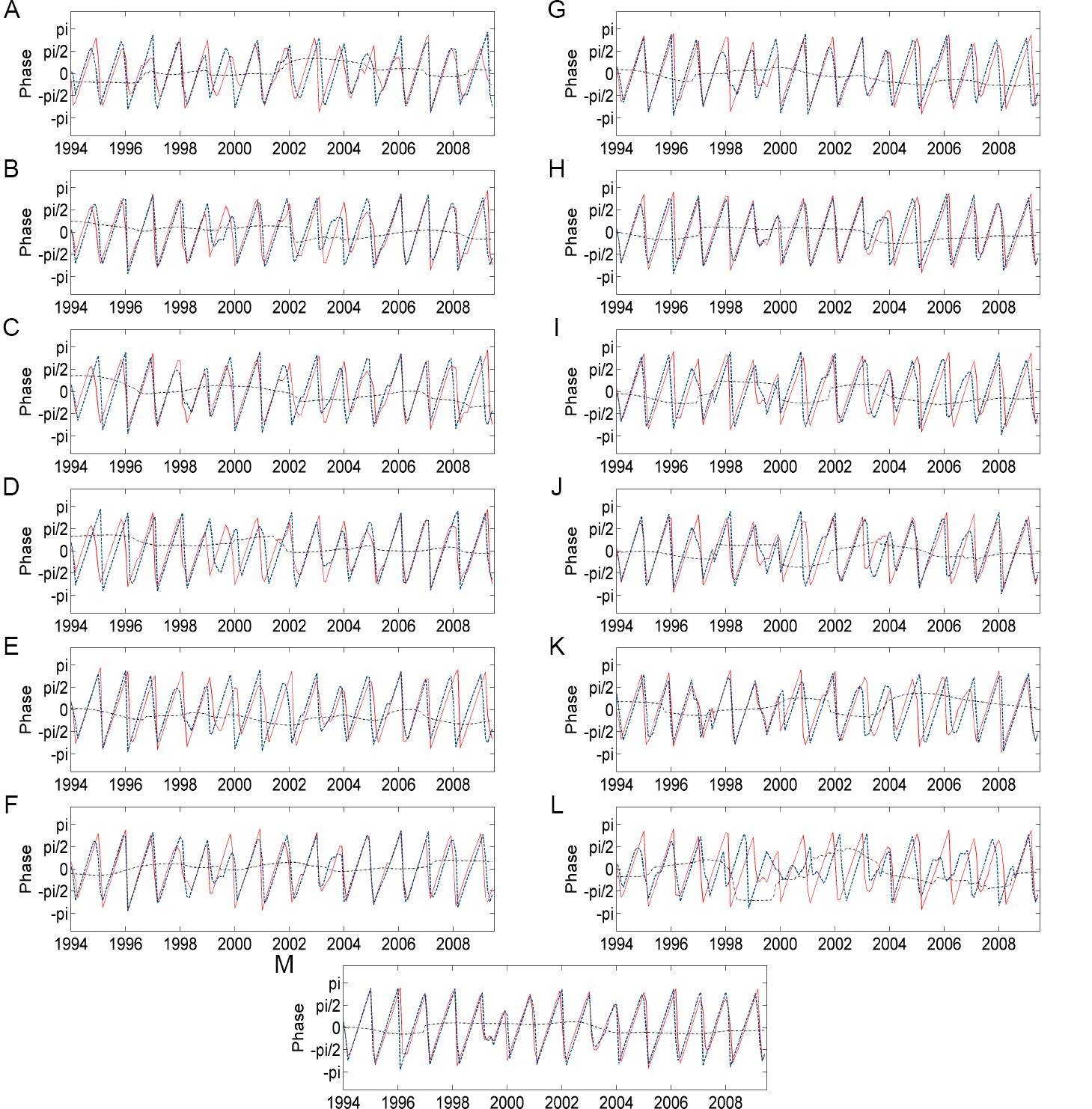

Supplement: Figure S2 — Phase analyses of dengue time series between neighboring districts in Binh Thuan province. Phase analyses between two districts (in blue and red), based on wavelets for 1-y periodic band. (1.21 MB TIF) [file pntd.0000747.s002.tif]
